# Management of extrusive luxation of upper incisors in young permanent teeth: a case report

**DOI:** 10.11604/pamj.2021.40.144.30656

**Published:** 2021-11-08

**Authors:** Emna Hidoussi Sakly

**Affiliations:** 1 Department of Restorative Dentistry-Endodontics, Oral Health and Oro-Facial Rehabilitation Laboratory Research, Faculty of Dental Medicine of Monastir, University of Monastir, Tunisia

**Keywords:** Dental trauma, tooth fracture, root canal therapy, case report

## Abstract

This case report documents the clinical approach adopted for two permanent maxillary incisors with extrusive luxation in a 16-year-old boy. The proposed procedures involved reposition of both teeth, by digital pressure and stabilized by using semi-rigid splint for 2 weeks. Endodontic therapy was performed. Clinical and radiographic follow-up examinations were conducted at 6-month intervals for two years. Assessment revealed the absence of pulpal and periapical disease and the restoration of the maxillary incisors to a state of health and normal function. A long-term clinical and radiological follow-up is needed to prevent and precociously detect possible complications that may occur following a extrusive luxation.

## Introduction

Traumatic dental injuries of permanent teeth are a public health problem due to high prevalence, especially among children and teenagers [1]. When the lesions affect the supporting tissues of the tooth, they lead to a dislocation trauma which is classified according to Andreansen as 6 types [2]. Among these lesions, extrusive luxations are an uncommon type of dental injury in the permanent dentition. The frequency has been found to be 7 and 11% among traumatized permanent teeth [3]. They mostly affects maxillary incisors during physical activities and may have functional, aesthetic and emotional repercussions [4]. This traumatic dental injury is characterized by a partial displacement of the tooth out if its socket. Due to the periodontal ligament injury, the luxated tooth has to be repositioned gently as soon as possible [5]. According to the current guidelines, a luxated tooth should be repositioned to its original position using digital pressure and stabilization for a minimum period of two weeks in order to favor periodontal ligament repair [6]. Endodontic treatment with calcium hydroxide should be placed to prevent root resorptions. However, the dental pulps of severely luxated immature teeth have a much higher rate of survival than those of mature teeth. Hence, pulp retention is recommended for severely luxated immature teeth whereas there is a lower occurrence rate of pulp necrosis thanks to the presence of a rich vascular blood supply to the pulp through a wide open apical foramen that preserves pulpal circulation. This article discusses a clinical case of dental trauma, following a sporting activity, involving extrusive luxation of maxillary permanent central incisors in a 16-year-old boy. The clinical approach involved repositioning teeth, an endodontic treatment, and two years of follow-up.

## Patient and observation

**Patient information:** a 16-year-old boy in a good general health was referred to the Dental Medicine Clinic of Monastir after a fall that resulted in dental trauma. The medical history was unremarkable.

**Figure 1:**
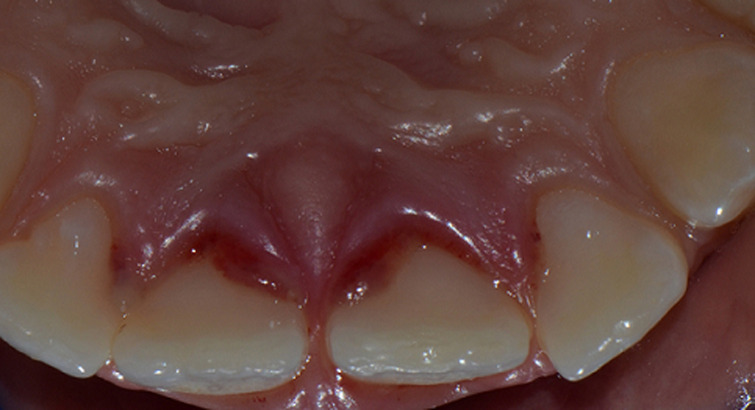
palatal clinical preoperative view of traumatized teeth

**Clinical findings:** the extraoral examination showed no other injuries of the lip or nose. Concerning the intraoral examination, the maxillary permanent central incisors appeared elongated with bleeding from the periodontal ligament. From a palatal and lateral view, it was observed that the crown of both central incisors was displaced to the palatal direction (Figure 1).

**Timeline of current episode:** October 2018: patient referral to the department of conservative dentistry and endodontics at the Dental Medicine Clinic of Monastir; November 2018: removal of the semi-rigid plint and endodontic treatment; January 2019: clinical control; April 2019: radiographic follow-up at 3 months; Novembre 2020: radiographic follow-up at 2 years.

**Figure 2:**
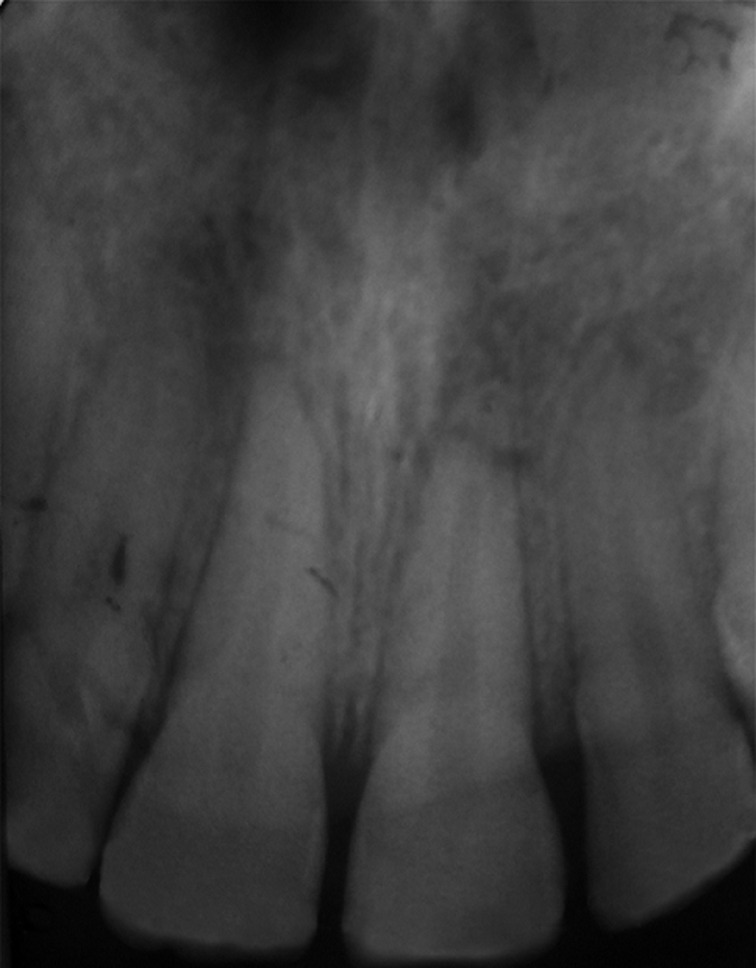
re-operative radiograph

**Diagnostic assessment:** the teeth were dislocated at a one-millimeter palatal version. Both maxillary central incisors were sensitive to percussion and exhibited class I tooth mobility. A periapical radiograph revealed that both incisors had closed apices, and no root or bone fractures were detected (Figure 2). We observed an increase in periodontal ligament space.

**Diagnosis:** the clinical diagnosis revealed an extrusive luxation of both maxillary central incisors.

**Figure 3:**
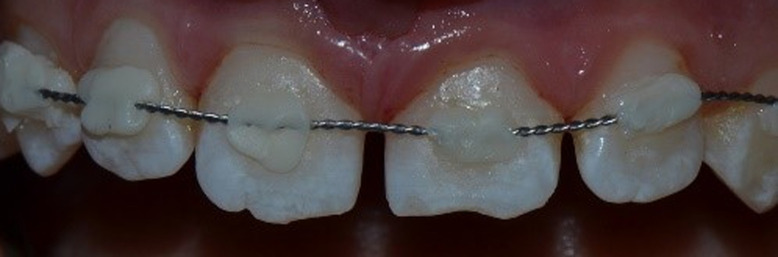
flexible splint made of resin composite and 0.7mm orthodontic wire onto the labial surface of the teeth involved and the immediately adjacent teeth

**Figure 4:**
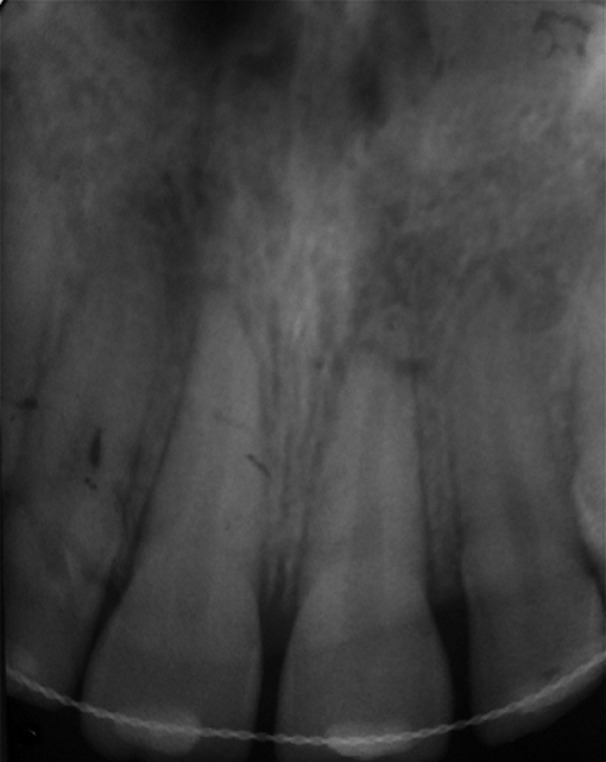
radiograph of splinted maxillary incisors immediately after injury

**Therapeutic interventions:** the emergency treatment consisted in repositioning the displaced teeth under local anesthesia. Both central incisors were repositioned by digital pressure. During repositioning, holding the luxated tooth by the incisal and palatal side of the crown is of extreme importance. The pushing force should be focused on the apex and directed simultaneously downwards and inwards. The teeth were correctly repositioned and the bone was gently set into the socket. The maxillary central incisors were then splinted to the adjacent laterals with a splint made of resin composite (Filtek Z350 XT, 3M ESPE) and 0.7mm orthodontic wire was placed on to the labial surface of the teeth. The teeth were stabilized for two weeks (Figure 3) and a periapical radiograph was taken (Figure 4). We stressed the importance of oral hygiene to the patient and prescribed chlorhexidine solution (0.12%) to apply twice daily on the injured region for a week after the dental trauma. In addition, paracetamol was administered to reduce post intervention pain.

**Figure 5:**
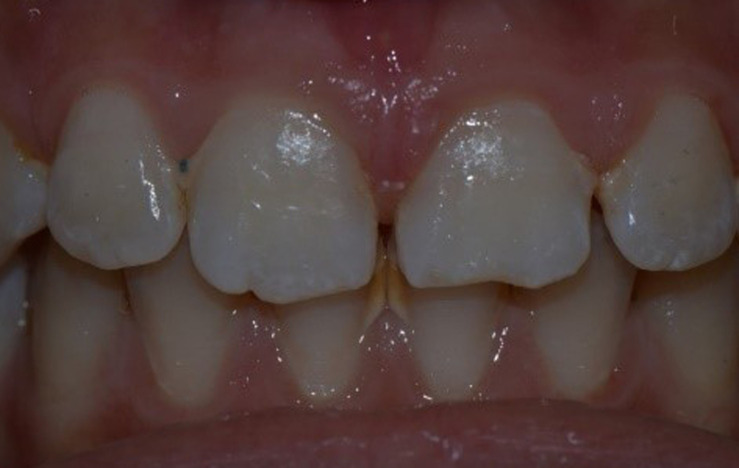
clinical aspect after removal of the splint showing the normality of soft tissues

**Figure 6:**
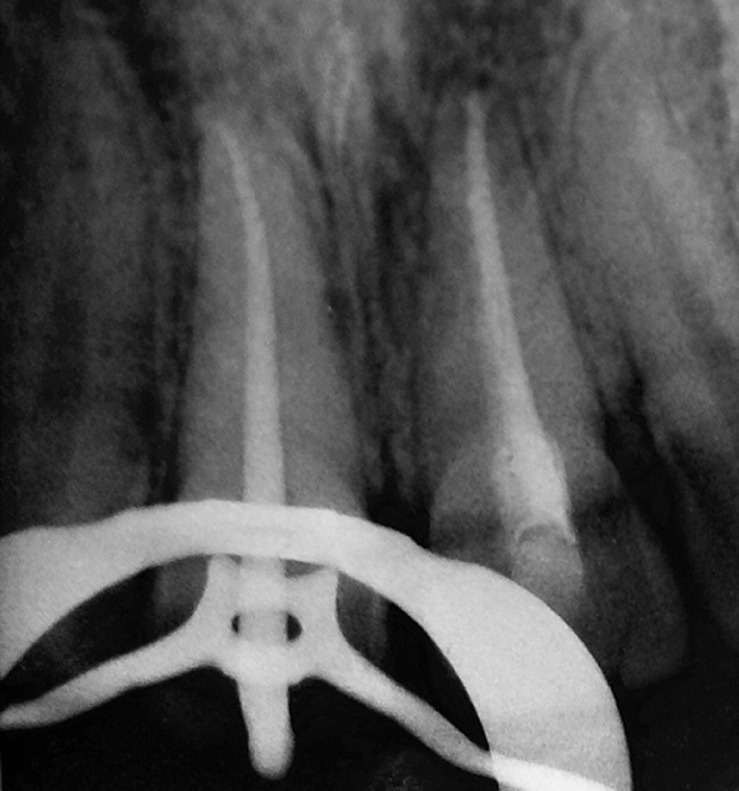
postobturation X-ray

**Figure 7:**
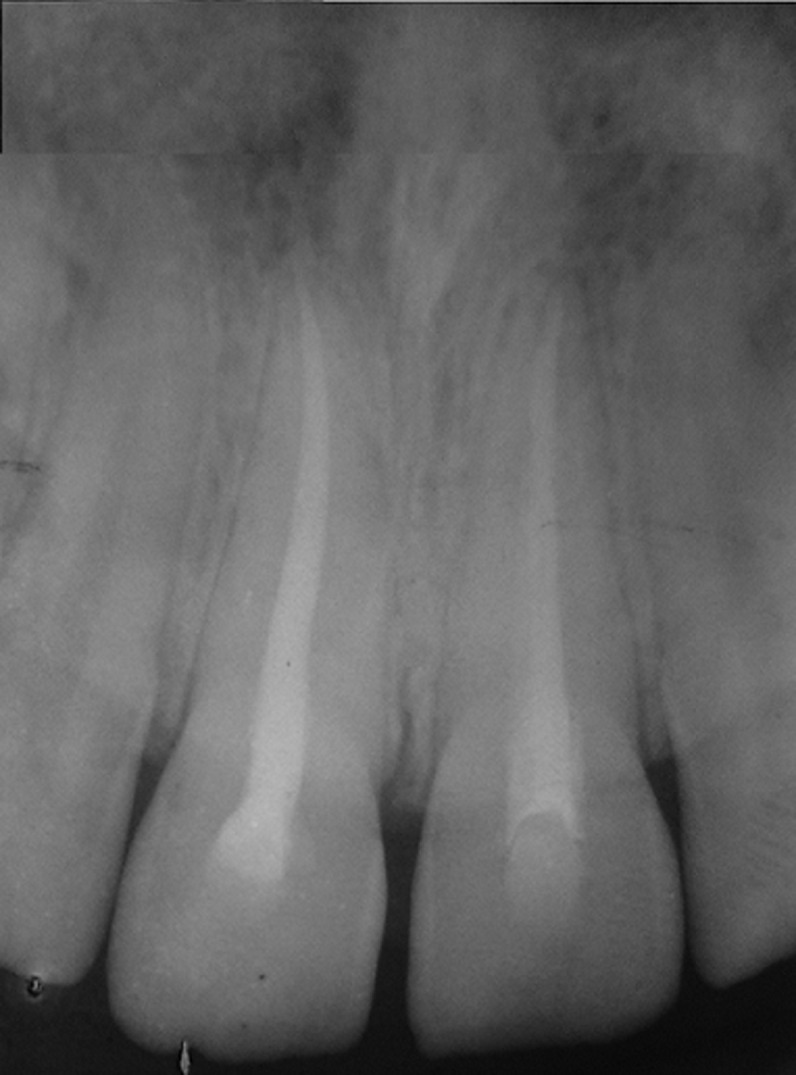
radiograph of maxillary incisors 2 years after injury

**Follow-up and outcome of interventions:** two weeks later, the mobility of the teeth was within the normal limits. Therefore, we removed the semi-rigid splint (Figure 5). During this first check-up appointment, the patient suffered a chewing-related pain. In addition, the teeth were sensitive to percussion. Therefore, in order to prevent root resorptions, endodontic treatment was performed for both maxillary central incisors. Under rubber dam, the access cavity was prepared and root canals were cleaned and shaped using nickel titanium instruments (2Shape, Micro Mega). Furthermore, the root canal was irrigated with 3mL of 2.5% sodium hypochlorite at each instrument change. The working lengths were determined using electronic apex locator (Rootor, META BIOMED) and controlled radiographically. The root canal was treated with calcium hydroxide (Ultracal, Ultradent Products, Inc., South Jordan, UT, USA). Then, we sealed the access cavity of the luxated teeth with temporary filling (MD-TempTM, META Biomed co). Three weeks later, the root canal was obturated with bioceramic sealer (BioRoot RCS, Septodont) and gutta-percha points (Figure 6). The access cavity was filled with composite resin (Filtek Z350 XT, 3M ESPE, St. Paul, MN, USA). Three months after the trauma, the patient´s teeth were asymptomatic. The patient felt no pain and teeth were insensitive to percussion. The gingival attachments were intact and the alveolar mucosa was normal without pain or tenderness to palpation. Appointments for subsequent follow-up examinations and dental cleanings were scheduled on a 6-month post-injury basis. After 2 years of follow-up, clinical and radiographic examinations revealed that the teeth were stable with normal mobility and no signs of root resorption and radiolucent image at the apex of the teeth (Figure 7).

**Patient perspective:** “I feel good that my tooth was conserved and I will continue clinical follow-up”.

**Informed consent:** the patient gave informed consent.

## Discussion

Dentoalveolar traumas are relatively frequent accidents and dramatic episodes can occur. The incidence of trauma involving permanent teeth is reported to be between 7% and 19% [7]. Among these traumas, extrusive luxations of teeth are a common type of dental injury in the permanent dentition and usually concerned the maxillary teeth. These teeth are most exposed to trauma due to their position on the bumper of the middle level of the face. Males, be they children or adults, are more prone to permanent teeth luxation. They spend more time doing school or sports activities and fighting than females. Collisions, falls and contact with hard surfaces during sports activities have an associated risk of orofacial injuries. Thus the American Academy of Pediatric Dentistry recommends using protective gears, including mouthguards, which help distribute forces of impact, thereby reducing the risk of severe injury [8]. In our clinical case, wearing a protective splint could have protected the patient against such accident. The recommended treatment is repositioning the teeth as soon as possible, into their normal position and stabilizing them with a splint for two weeks. The repositioning must be carried out by forcing the displaced apex out of its locked position within the labial bone and then applying axial pressure in apical direction to manipulate the tooth into its natural position. If complete repositioning is not possible due to a delayed treatment, other treatment options should be planned, such as intentional replantation, taking into consideration a technique that presents less risk of damage to the root surface [9]. Stabilizing an injured tooth with the help of its neighboring sound teeth is an excellent practice.

This practice not only reduces pain, but also provides comfort to the patient and protects the fixed tooth against further physiological forces [10]. The splint made from orthodontic wire and resin composite to stabilize traumatically displaced teeth, as performed in the present case, has the advantage of using low-cost materials generally available in dental offices. Also it leads to satisfactory outcomes because the characteristics decrease the risks of complications such as ankylosis, root resorption, and pulp obliteration [10]. Flexible wire-composite splints could also be used for longer periods until the healing of the periodontal ligament. Depending on the severity of tooth displacement, luxation may lead to contusion, elongation or tearing of the vasculo-nervous bundle in the apex region. Further, in the most favourable cases, a temporary reduction in the blood flow to the pulp may result. This phenomenon, however, tends to heal completely within a few weeks. A complete rupture of the vasculo-nervous bundle will most often lead to a pulp necrosis. It is only on young teeth with a widely open apex that a revascularization is likely. A three-part study was carried out on 144 severely luxated permanent incisors in patients aged between 6 and 18 years with an average age of 10 years, who were followed over a mean period of 3.8 years.

The results showed that the incidence rates were 43% for pulp necrosis, 34% for pulp survival and 23% for pulp calcification [11]. Nevertheless, the pulp of luxated immature teeth showed a much higher rate of survival and a much lower rate of necrosis than those of mature teeth with each sort of dislocated injury. Contrarily, pulp necrosis is frequent in extrusive luxation of permanent teeth with closed apices. Endodontic treatment with calcium hydroxide is essential for the prevention of subsequent infection-related resorption. It exerts effective inflammatory resorption control. According to the literature, the alkaline pH of calcium hydroxide plays an antimicrobial role in the root canal and blocks the evolution of infectious processes. Additionally, toxins produced by bacteria are denatured by the action of calcium hydroxide. Therefore, in our clinical case, we carried out an endodontic treatment after two weeks and placed an intra canal medication based on calcium hydroxide. The endodontic treatment of the traumatized teeth aimed to reduce the consequences of pulp necrosis, such as periapical lesions and particularly, external root resorptions of infectious origin as much as possible. The traditional protocol involves trepanning the teeth within 7-14 days after the trauma followed by mechanical root preparation. Calcium hydroxide serves as a drug for the treatment of infected root canals [1]. However, calcium hydroxide has no effect on the initial inflammatory reaction taking place at the periodontium of all traumatized teeth. Nevertheless, the severity of this initial inflammatory reaction as well as the lesions of the root cement is crucial for the subsequent healing pattern of periodontal tissues.

## Conclusion

Extrusive luxation is a serious dental trauma that involves the periodontal complex. A correct diagnosis followed by repositioning the tooth in its initial position is fundamental for the healing of the periodontium. In the case of permanent teeth with closed apices, the preventive endodontic treatment using a dressing of calcium hydroxide resulted in a positive alternative. This reported case could be considered a success because both teeth were preserved during 2 years without any symptoms, with perfect retention and function.
